# Clinical and Procedural Characteristics of Patients Undergoing Endobronchial Ultrasound-Guided Transbronchial Mediastinal Cryobiopsy (CryoEBUS): A Descriptive Study in a Colombian Population

**DOI:** 10.7759/cureus.89253

**Published:** 2025-08-02

**Authors:** Jacobo Echeverri-Hoyos, Jaime A Echeverri Franco, Eduardo Tuta-Quintero

**Affiliations:** 1 School of Medicine, Institución Universitaria Visión de las Américas, Pereira, COL; 2 Pulmonology, Clínica de Alta Tecnología Oncólogos del Occidente, Pereira, COL; 3 Epidemiology and Public Health, Universidad de La Sabana, Chía, COL

**Keywords:** cryobiopsy, diagnosis, endobronchial ultrasound, mediastinal lesion, safety

## Abstract

Endobronchial ultrasound-guided transbronchial mediastinal cryobiopsy (CryoEBUS) is an emerging minimally invasive technique that provides larger, better-preserved samples compared to conventional needle aspiration, improving suitability for histopathological and molecular testing. This retrospective study aimed to characterize the clinical profile, procedural aspects, and diagnostic yield of CryoEBUS in patients undergoing biopsy for mediastinal lesions. Fifty-nine patients underwent CryoEBUS. The mean age was 64.4 years (SD: 15.8), and 54% were men. Systemic arterial hypertension was the most common comorbidity (34%). The most frequent presenting symptoms were cough (66.1%) and chest pain (32.2%). CryoEBUS was performed for mediastinal lesion sampling, with an average of 1.5 lymph node stations biopsied per patient. Station selection was based on imaging characteristics (size ≥10 mm or fluorodeoxyglucose (FDG) uptake). Freeze time ranged from four to six seconds. Histopathology confirmed malignancy in 76% of cases, with adenocarcinoma being the most common subtype (42%). Diagnostic success, defined as the acquisition of a conclusive histopathological diagnosis, was achieved in 93% of cases. Minor complications occurred in 17%, primarily self-limited bleeding. CryoEBUS appears to be a safe and effective diagnostic tool for mediastinal lesion evaluation. Its ability to provide high-quality tissue samples supports its clinical utility, especially when molecular or immunohistochemical studies are required for diagnosis and treatment planning.

## Introduction

Endobronchial ultrasound-guided transbronchial needle aspiration (EBUS-TBNA) is widely recognized as the preferred technique for evaluating mediastinal lymphadenopathy and staging non-small cell lung cancer (NSCLC), owing to its minimally invasive nature and established diagnostic accuracy [1,2]. However, its limitations in providing sufficient tissue for certain histological and molecular analyses have prompted the exploration of complementary approaches [1,3]. One such technique is endobronchial ultrasound-guided transbronchial mediastinal cryobiopsy, also referred to as CryoEBUS, which has emerged as a promising alternative to improve tissue acquisition in selected mediastinal pathologies [1,2].

CryoEBUS allows for the retrieval of larger, well-preserved samples that are typically less prone to artifacts such as hemorrhage or crush distortion [1,2,4]. This may be particularly advantageous in the diagnosis of lymphoproliferative diseases, rare tumors, and benign conditions where architectural integrity is essential [1,2,4,5]. Furthermore, the improved sample quality can facilitate comprehensive molecular and immunohistochemical profiling in patients with suspected or confirmed lung cancer [3,5].

While the technique is still evolving, recent studies support its favorable safety and diagnostic performance [3,6]. For instance, Zhang et al. [7] reported a significantly higher diagnostic yield with CryoEBUS compared to EBUS-TBNA (91.8% vs. 79.9%; p = 0.001), particularly in uncommon tumors (sensitivity 91.7% vs. 25%; p = 0.001) and benign diseases (80.9% vs. 53.2%; p = 0.004). Despite these promising results, challenges remain regarding standardization, procedural protocols, and broader clinical implementation [6].

Given the growing body of evidence and the potential benefits in diagnostic accuracy, especially in resource-limited settings, further characterization of CryoEBUS use in real-world practice is warranted. Therefore, the objective of this study was to describe the clinical and procedural characteristics of patients undergoing mediastinal lymph node or mass biopsy using CryoEBUS in a Colombian population.

## Materials and methods

Study design and population

We conducted an observational study in patients undergoing lymph node or nodule/mass biopsy using CryoEBUS. The study was conducted at Oncólogos del Occidente S.A.S. (Pereira, Colombia) after obtaining approval from the Comité de Ética - Investigación de Oncólogos del Occidente S.A.S. (approval number: CÓDIGO RG-FO-001). Adults over 18 years of age with clinical and radiological suspicion of neoplastic, infectious, or other diseases were included. Patients with contraindications to the procedure or incomplete clinical records were excluded.

Variables and data collection

Collected data included demographic variables (age and sex), presence of comorbidities, and clinical symptoms such as cough, chest pain, weight loss, profuse diaphoresis, and dyspnea. Procedures were categorized as lymph node biopsy or nodule/mass biopsy based on the clinical indication. The American Society of Anesthesiologists (ASA) physical status classification was recorded for each patient prior to the procedure.

All samples were evaluated by experienced pathologists. Diagnoses were classified as malignant, infectious (e.g., tuberculosis or cryptococcosis), sarcoidosis, or non-diagnostic. For malignant cases, histological subtypes such as adenocarcinoma, squamous cell carcinoma, and small cell carcinoma were specified. The use of positron emission tomography-computed tomography (PET-CT) and the presence of hypermetabolic lymph nodes were recorded, along with the average number of such nodes per patient. Molecular studies were performed when clinically indicated.

To reduce potential sources of bias, patients were included consecutively to avoid selection bias. All clinical data, including symptoms and comorbidities, were extracted from standardized electronic medical records by trained personnel using a predefined data collection form. Histopathological analyses were performed by experienced pathologists blinded to the clinical and radiological context to reduce diagnostic bias. Additionally, the procedures were carried out by trained bronchoscopists following standardized protocols to ensure consistency in sample acquisition.

Procedure and histopathological diagnosis

TBNA was performed using a Boston Scientific Expect™ pulmonary needle (Marlborough, MA, USA) under EBUS guidance. Tunnelization with the same needle was followed by tissue sampling with a 1.1 mm disposable flexible cryoprobe (ERBECRYO2) during the same procedure. Samples were obtained from lymph nodes and mediastinal lesions in contact with the central airway, with freeze times of 4-5 seconds. The mean number and anatomical distribution of sampled stations were recorded. Ultrasound characteristics suggestive of malignancy or benignity were also documented, including lesion size, shape, echogenicity, presence of calcifications, central necrosis, hilar structure visibility, and margin definition.

Statistical analysis

Quantitative variables were expressed as means and standard deviations, while categorical variables were summarized as frequencies and percentages. Statistical analyses were performed using Microsoft Excel 2017 (Microsoft Corporation, Redmond, WA, USA) and Stata version 17.0 (StataCorp LLC, College Station, TX, USA).

## Results

A total of 59 patients were included in the study, of whom 54% (32/59) were male, with a mean age of 64.4 years (SD: 15.8) (Table 1). The most common comorbidities were non-metastatic tumors (54%, 32/59) and systemic arterial hypertension (34%, 20/59), with an average of 2.97 comorbidities per patient (SD: 1.7). According to the ASA physical status classification, 61% (36/59) of patients were classified as ASA III.

**Table 1:** General characteristics of the population SD: standard deviation

Men, n (%)	32 (54)
Age years, mean (SD)	64.4 (15.8)
Comorbidities, n (%)	
Coronary artery disease	13 (22)
Chronic heart failure	7 (12)
Peripheral vascular disease	3 (5)
Systemic arterial hypertension	20 (34)
Alcoholism	4 (9)
Thromboembolic disease	5 (8)
Arrhythmia	2 (3)
Chronic obstructive pulmonary disease	19 (32)
Connective tissue disease	3 (5)
Kidney disease	8 (14)
Diabetes mellitus	4 (7)
Tumors without metastasis	32 (54)
Lymphoma	22 (37)
Solid tumor with metastasis	7 (12)
Human immunodeficiency virus infection	9 (15)
Number of comorbidities, mean (SD)	2.97 (1.7)

The most frequently reported symptoms prompting the procedure were cough (66%, 39/59), chest pain (32%, 19/59), and dyspnea (27%, 16/59) (Table 2). In all cases (100%, 59/59), the indication for CryoEBUS was the biopsy of a mediastinal lesion.

**Table 2:** Clinical features and pre-procedure evaluation ASA: American Society of Anesthesiologists

Pre-procedure symptoms, n (%)	
Cough	39 (66)
Chest pain	19 (32)
Weight loss	15 (25)
Profuse sweating	9 (15)
Dyspnea	16 (27)
Indication for the procedure, n (%)	
Mediastinal lesion biopsy	59 (100)
ASA score, n (%)	
2	18 (30)
3	36 (61)
4	5 (9)

Procedurally, the mean number of punctured stations per patient was 1.5 (SD: 0.8), with station 7 being the most commonly sampled (78%, 46/59) (Table 3). The average lesion size was 20.1 mm (SD: 9). Ultrasound features suggestive of malignancy were observed in 68% (40/59) of cases, while 32% (19/59) showed characteristics consistent with benign lesions. Among the ultrasound findings, 64% (38/59) had well-defined margins, 54% (32/59) were round, 73% (43/59) showed heterogeneous echogenicity, and 61% (36/59) lacked a central hilar structure. Post-procedure bronchospasm occurred in 14% (8/59) of patients.

**Table 3:** Injury characteristics, procedures, and complications SD: standard deviation

Number of stations punctured, mean (SD)	1.5 (0.8)
Stations punctured, n (%)	
11L	4 (9)
10L	5 (8)
4L	4 (7)
2L	2 (3)
7	46 (78)
2R	3 (5)
4R	15 (25)
10R	7 (12)
11Rs	3 (5)
Lesion sizes mm, mean (SD)	20.1 (9)
Node-lesion characteristics suggestive of malignancy	40 (68)
Node-lesion characteristics suggestive of benignity	19 (32)
Oval or elongated	27 (46)
Rounded	32 (54)
Distinct margins	38 (64)
Heterogeneous echogenicity	43 (73)
Calcifications	14 (24)
Central necrosis	23 (39)
Absent hilum	36 (61)
Complications, n (%)	
None	49 (83)
Bronchospasm	8 (14)
Infections	2 (3)

Histopathological analysis revealed malignancy in 76% (45/59) of cases, while 17% (10/59) were negative for malignancy and 7% (4/59) were classified as non-diagnostic (Table 4). Adenocarcinoma was the most frequent malignant subtype, observed in 42% (25/59) of patients. PET-CT was performed in 19% (11/59) of patients, with hypermetabolic lymph nodes detected in 91% (10/11) of them. Molecular studies were conducted in 12% (7/59) of patients, all of whom had a confirmed malignant diagnosis.

**Table 4:** Histological diagnosis, molecular characterization, and findings in chest imaging SD: standard deviation; PET-CT: positron emission tomography-computed tomography; PD-L1: programmed cell death receptor ligand-1; ALK: anaplastic lymphoma kinase; KRAS: Kirsten rat sarcoma viral oncogene homolog; BRAF: v-Raf murine sarcoma viral oncogene homolog B1

Histological diagnosis, n (%)	
Negative for malignancy	10 (17)
Positive for malignancy	45 (64)
Cryptococcosis	1 (2)
Tuberculosis	7 (12)
Sarcoidosis	2 (3)
Inconclusive	4 (7)
Malignancy histology, n (%)	
Squamous cell carcinoma	13 (22)
Adenocarcinoma	25 (42)
Small cell carcinoma	3 (5)
Lymphomas	4 (7)
PET-CT, n (%)	11 (19)
Hyperenhanced lymph nodes	10 (91)
Number of lymph nodes with increased uptake, mean (SD)	1.8 (1)
Molecular studies, n (%)	7 (100)
PD-L1 >1%, n (%)	3 (43)
KRAS positive, n (%)	1 (14)
ALK positive, n (%)	2 (29)
BRAF positive, n (%)	1 (14)

## Discussion

This study describes the clinical characteristics, procedural indications, and outcomes associated with the use of CryoEBUS for lymph node and mediastinal nodule/mass biopsy in a cohort of 59 patients. The majority of patients (54%) were male, with a mean age of 64.4 years, and the most prevalent comorbidities were non-metastatic tumors and hypertension. Cough was the most commonly reported symptom prompting the procedure, and biopsy of a mediastinal lesion was the indication in all cases. Station 7 was the most frequently sampled, and ultrasound findings commonly suggested malignancy, including features such as heterogeneous echogenicity, round shape, and absence of a hilar structure. Histological analysis confirmed malignancy in 76% of patients, with adenocarcinoma as the most frequent subtype. Non-diagnostic samples were obtained in 7% of cases. These findings contribute to refining the clinical profile of candidates for CryoEBUS and emphasize its value as a diagnostic tool, particularly in patients with suspected mediastinal malignancy.

Although this was a purely descriptive study without hypothesis testing, the high diagnostic yield for malignancy and the low rate of non-diagnostic samples support the diagnostic effectiveness of CryoEBUS. Previous reports have described diagnostic yields of up to 85% and pneumothorax rates around 4.2% [8]. While our study design does not allow for direct comparison with other techniques, our findings align with those reported for conventional EBUS-TBNA, CT-guided transthoracic biopsy, and mediastinoscopy, suggesting that CryoEBUS offers comparable diagnostic accuracy with an acceptable safety profile [8-11]. The relatively high malignancy detection rate observed in our cohort reinforces the potential utility of this approach. However, the presence of non-diagnostic samples in 7% of cases highlights the need to optimize sampling techniques and explore adjunctive strategies to improve diagnostic yield.

Comorbidities are critical considerations when selecting patients for diagnostic procedures, especially among elderly individuals or those with chronic illnesses who may benefit from less invasive approaches [12-14]. In our cohort, there was a high prevalence of comorbidities, particularly non-metastatic malignancies and hypertension. This is consistent with previous studies showing that patients undergoing mediastinal diagnostic procedures often have multiple comorbid conditions, which may increase the risk of complications and limit the feasibility of more invasive diagnostic methods [12,14].

The clinical presentation in our cohort, notably symptoms such as cough, dyspnea, and chest pain, supports the timely indication of diagnostic procedures like CryoEBUS in patients with suspected intrathoracic disease [11-13]. Among patients diagnosed with malignancy, 64% of lesions showed ultrasound features commonly associated with neoplastic disease. Future studies should assess whether specific ultrasound characteristics correlate with diagnostic accuracy and malignancy risk, to better guide clinical decision-making.

The limited number of molecular tests performed in this study restricts our ability to evaluate the full potential of CryoEBUS in providing adequate tissue for molecular profiling. Molecular characterization plays a central role in treatment selection, especially in lung adenocarcinoma and other thoracic malignancies with actionable genetic mutations [15,16]. Although previous studies have shown that cryobiopsy samples are suitable for molecular analysis, further research is needed to validate the adequacy of CryoEBUS-obtained specimens for advanced diagnostics [15,16].

Importantly, no cases of bleeding or pneumothorax were reported in our cohort. This may reflect the use of a standardized and cautious technique performed by experienced operators, including Doppler ultrasound, to evaluate vascularity with radial EBUS when needed. These findings further support the safety profile of CryoEBUS when performed under appropriate procedural conditions [17].

This study has several limitations inherent to its observational, single-center design. The findings may not be generalizable to other settings with different patient populations, healthcare infrastructure, or operator expertise. Additionally, the retrospective nature of data collection introduces the potential for information bias, particularly regarding symptoms or clinical variables not consistently documented. Selection bias may also be present, as the decision to perform CryoEBUS was made by the treating physicians and may have favored patients with radiologic or clinical suspicion of malignancy. To validate and extend our findings, larger prospective multicenter studies with standardized data collection and long-term follow-up are needed.

## Conclusions

CryoEBUS demonstrated a diagnostic yield of 76% for malignancy in patients undergoing biopsy of mediastinal lymph nodes and masses, with adenocarcinoma being the most common histologic subtype. The procedure showed a favorable safety profile, with a 14% incidence of bronchospasm and no cases of pneumothorax or bleeding. Most patients had comorbid conditions such as non-metastatic cancer and hypertension, and station 7 was the most frequently sampled site.

Despite these strengths, a non-diagnostic rate of 7% highlights areas for improvement. Potential contributing factors may include lesion characteristics, sampling technique, or operator experience. Strategies such as enhanced training, protocol standardization, and the use of adjunctive imaging tools may help reduce the rate of inconclusive results and improve tissue adequacy.

Future prospective multicenter studies are needed to validate these findings, particularly regarding the feasibility of molecular testing with CryoEBUS-obtained samples, its cost-effectiveness, and long-term clinical outcomes in diverse patient populations.
